# Modeling the Association Between Repeated Measures of Hemoglobin During Pregnancy and Adverse Birth Outcomes

**DOI:** 10.1016/j.tjnut.2026.101511

**Published:** 2026-03-29

**Authors:** Jiaxi Geng, Ziwei Zhang, Phuong Hong Nguyen, Hanqi Luo, Melissa F Young, Yi-An Ko

**Affiliations:** 1 Department of Biostatistics and Bioinformatics, Rollins School of Public Health, Emory University, Atlanta, GA, USA; 2 Nutrition, Diets, and Health Unit, International Food Policy Research Institute (IFPRI), Washington D.C., USA; 3 Hubert Department of Global Health, Rollins School of Public Health, Emory University, Atlanta, GA, USA

**Keywords:** Maternal hemoglobin, Birth outcomes, Longitudinal analysis, Distributed lag model, Generalized additive mixed model, Group-based trajectory modeling, Mixed-effects model

## Abstract

**Background:**

Maternal hemoglobin (Hb) concentrations and their trajectories throughout pregnancy are important determinants of birth outcomes. However, longitudinal studies on pregnancy frequently rely on cross-sectional analyses at specific time points or utilize summary measures, overlooking valuable information contained in repeated Hb measurements.

**Objective:**

To illustrate various statistical approaches for modeling longitudinal Hb data and their associations with birth outcomes, highlighting the strengths and limitations of each method.

**Methods:**

We analyzed 8 pregnancy datasets (6,452 women, 13,580 Hb measurements) from the Biomarker Reflecting Inflammation and Nutritional Determinants of Anemia (BRINDA) project using: 1) logistic regression incorporating residual Hb, 2) two-stage mixed effect model, 3) distributed lag non-linear model (DLNM), 4) generalized additive mixed model (GAMM), and 5) group-based trajectory modeling (GBTM). Outcomes were low birth weight (LBW, < 2.5kg), preterm birth (PTB, < 37 weeks), and small for gestational age (SGA, birthweight < 10th percentile).

**Results:**

Logistic regression using residual Hb, two-stage mixed-effects model, and DLNM did not reveal any significant associations between Hb concentrations and adverse birth outcomes. GAMM showed that women with PTB had lower Hb concentrations before 20 weeks of gestation compared to those with without PTB. GBTM identified four distinct Hb trajectory clusters, but no significant associations were found between trajectory groups and adverse birth outcomes.

**Conclusions:**

These analytic approaches provide complementary insights into the relationship between maternal Hb and birth outcomes, while illustrating how inference can vary depending on the method used. DLNMs can help pinpoint critical gestational periods of vulnerability, while models such as GAMM and GBTM capture nonlinear trends and heterogeneous trajectories. Researchers should be aware that conclusions about hemoglobin and birth outcomes may be highly sensitive to modeling decisions. Overall, these methods can guide researchers in selecting statistical strategies best suited to their study aims and data structure.

## Introduction

The nutritional status of pregnant women plays a crucial role in maternal health, fetal and newborn growth and development ^1,2^. Hemoglobin (Hb), a protein in red blood cells, is vital for transporting oxygen from the lungs to tissues throughout the body ^3^. A decline in Hb production reduces the number of red blood cells, thereby impairing oxygen delivery to the body’s cells ^4^. During pregnancy, both abnormally high and low Hb concentrations have been linked to impaired fetal development and adverse birth outcomes ^5^. For example, one study found that maternal Hb concentration exceeding 130 g/L in the third trimester was associated with an increased risk of adverse birth outcome, whereas lower Hb concentrations were related with a decreased risk of preterm birth (PTB) and small for gestational age (SGA) ^6^. Conversely, another study reported that pregnant women with low Hb concentrations in early pregnancy had significantly higher risks of PTB and low birth weight (LBW) ^7^. These findings underscore the importance of maintaining optimal hemoglobin levels throughout pregnancy to support proper fetal development. Because Hb concentrations fluctuate during the gestation ^8^, analyzing longitudinal measures of Hb can provide a more comprehensive understanding of how Hb changes affect pregnancy outcomes over time.

In pregnancy research, repeated measurements of biomarkers are commonly collected to monitor changes over time. For example, studies on iron and folic acid supplementation among pregnant women rely on multiple antenatal care visits to track maternal health ^9,10^. Evidence suggests that the association between Hb concentrations and birth outcomes varies by trimester, indicating that changes in Hb at specific gestational periods may be particularly relevant ^11^. Common analytical approaches include regressing the dependent variable on Hb concentrations categorized into groups ^6,12,13^ or on Hb trajectory groups ^14^. While these projects attempt to summarize longitudinal data but often fail to fully capture the dynamic, time-varying relationship between repeated maternal biomarker measurements and adverse birth outcomes. There remains a notable gap in nutrition research regarding systematic evaluations of statistical methods that model repeated maternal biomarker measurements with respect to time-invariant outcomes. Specifically, it remains unclear that how different methods would capture Hb’s effect on adverse birth outcomes based on their unique statistical assumptions.

To address this gap, this study employs five established statistical methods tailored for modeling a time-varying predictor to investigate the association between repeated measurements of Hb and birth outcomes, highlighting the inherent advantages and limitations of each method. Unlike previous reviews, we highlighted the inference distinctions between different methods, showing the interpretation of each method. Researchers and data analysts can benefit from this study by gaining insights into selecting appropriate statistical approaches for handling repeated measures of an exposure variable with a binary outcome, thereby enhancing our understanding of the complex relationship between maternal nutrition and birth outcomes.

## Methods

### Study setting

The study included 8 datasets (6,452 pregnant women with 2 or 3 Hb measurements) from the Biomarkers Reflecting Inflammation and Nutritional Determinants of Anemia (BRINDA) project ^15^. They came from 7 countries, including Bangladesh^16,17^ (2012 data: N=1283; 2013 data: N=716), Ghana ^18^ (N=1044), Gambia ^19^ (N=363), Malawi ^20^ (N=1012), Mexico ^21^ (N=502), United States ^22^ (N=147), and Vietnam ^23^ (N=1385). All the studies were randomized trials, except for one cohort study ^22^.

### Hemoglobin measurements

Across the 8 datasets, maternal Hb was primarily measured using HemoCue systems. However, blood source varied by dataset: Hb was assessed using venous blood in Bangladesh (2012), Gambia, Ghana, Malawi, and the United States, and Capillary blood in Bangladesh (2013), Mexico, and Vietnam. Additionally, Hb concentration was adjusted for both altitude and smoking status in the Vietnam dataset. Figure S1 showed mean Hb concentrations by trimester across datasets.

### Birth outcomes

Birth outcomes were preterm birth (PTB), low birth weight (LBW), and small for gestational age (SGA). PTB was defined as delivery before 37 weeks of gestation. LBW was defined as a birth weight less than 2500 grams, PTB was defined as a gestational age at delivery less than 37 weeks, and SGA was defined as a birth weight less than the 10th percentile for gestational age according to the 21st Intergrowth guidance.^24^

### Notations

We use $Y_{i}$ to denote a binary outcome for subject i ($Y_{i}=0\;or\;1,\;i=1,\;...,\;N$), and $X_{ij}$ to denote the measurement of Hb at visit j for subject $i\;(j\;=\;1,\;\ldots ,\;n_{i}$, where $n_{i}$ is the number of Hb measurements for subject i), where $n_{i}=2$ or 3 in this application. Each visit occurs at time (gestational age) $t_{ij}$ in trimester $T_{ij}$ (e.g., $t_{ij}=30$ weeks in gestational age corresponds to the third trimester $T_{ij}=3$). Pregnancy trimester was categorized as: first trimester (0–13 weeks), second trimester (13–29 weeks), and third trimester (29–41 weeks).

### Statistical models for analyzing repeated hemoglobin measurements and birth outcomes

To analyze the relationship between repeated Hb measurements and birth outcomes, we applied 5 statistical models: logistic regression using residual Hb, two-stage mixed-effects model, distributed lag model, generalized additive mixed model, and group-based trajectory model. When participants had two Hb measurements collected within the same trimester, a deviation from the study protocol that likely reflected scheduling or clinic logistics rather than physiological change, we averaged these values within the trimester. This resulted in N=5776 with 2 measurements and N=676 with 3 measurements (in all three trimesters) of Hb. Generalized additive mixed models were applied to the full dataset (N = 6,452 women with 2 or 3 Hb measurements). The logistic regression, two-stage mixed-effects, distributed lag, and the group-based trajectory models were applied to a subset of women with 3 Hb measurements (N = 676).

#### Logistic regression using residuals

To avoid the issue of collinearity of having repeated Hb measurements simultaneously as regressors, one approach is to utilize residual Hb instead of the original value.^25^ This method involves two steps. First, a Hb measurement, measured at a later stage of pregnancy, is regressed on all the Hb measurements in previous visits using a linear regression. The residuals are then standardized to denote window-specific change in Hb values. This step is repeated for each Hb measurement collected after the first visit. Next, the first-visit Hb and all the residual Hb are included as predictors in a logistic regression to quantify relative importance of intra-trimester changes in Hb on a binary outcome. Essentially,

$$X_{i2}=\;a_{0}+a_{1}X_{i1}+\epsilon_{i1}$$

$$X_{i3}=\;a_{0}+a_{1}X_{i1}+a_{2}X_{i2}+\epsilon_{i2}$$

The second visit Hb ($X_{i2}$) was regressed on the first visit Hb ($X_{i1}$) using linear regression. The third visit Hb ($X_{i3}$) was regressed on the first visit Hb ($X_{i1}$) and the second visit Hb ($X_{i2}$) using a separate linear regression. The residuals from both models $({\hat{\epsilon }}_{i1}{and\;\hat{\epsilon }}_{i2}$) along with the first visit Hb ($X_{i1}$) were then used as covariates in the outcome logistic regression.


$$logit\left[P\left(Y_{i}=1\right)\right]=\beta_{0}+\beta_{1}X_{i1}+\beta_{2}{\hat{\epsilon }}_{i1}+\beta_{3}{\hat{\epsilon }}_{i2}$$

#### Two-stage mixed-effects model

The two-stage approach ^26^ allows for capturing the longitudinal trend of Hb, making it relevant for understanding the relationship between the changes in Hb and outcomes. It consists of the following two stages.


$$Stage\;1:\;X_{ij}=a_{0i}+a_{0}+a_{1i}t_{ij}+a_{1}t_{ij}+\epsilon_{ij},$$

$$Stage\;2:\;logit[P(Y_{i}=1)|a_{0i},a_{1i})]=\beta_{0}+{\beta_{1}\hat{a}}_{0i}+{\beta_{2}\hat{a}}_{1i}.$$

In Stage 1, a linear mixed model is used to model the relationship between Hb and time. $t_{ij}$ indicates time point (gestational age or trimester) and can be treated as either a continuous or a discrete variable. $a_{0i}$ and $a_{1i}$ are random intercept and random slope, which present how the individual-specific initial value and its change (per unit increase in time) differ from the population mean. $a_{0}$ and $a_{1}$ are fixed effects. $\epsilon_{ij}$ is the random error term, assumed to be normally distributed with mean 0. It is possible to include polynomial or spline terms of time to characterize non-linear relationships when multiple repeated measurements are available. In Stage 2, the estimated random effects are extracted from Stage 1 and used as predictors in the subsequent outcome logistic regression model. For the BRINDA pregnancy data analysis, we used trimester (2nd vs. 1st, 3rd vs. 1^st^) as fixed and random effects in Stage 1.

#### Distributed lag model

Distributed lag models (DLM) ^27^ are widely used in environmental exposure research to estimate the potentially delayed effects of time-varying exposures and to identify critical windows of susceptibility. The DLM models time series data using a regression framework that relates the outcome not only to the current exposure value but also to its lagged (past) values. Developing a DLM generally involves three main steps.

Step 1. Defining the exposure–response relationship. The first step is to define the functional form of the exposure–response association using basis functions. To characterize potentially nonlinear relationships between exposure and outcome, flexible functions such as splines, polynomials, or piecewise functions can be used. These functions transform the original exposure variable into a set of basis variables that describe the shape of the exposure–response curve.

Step 2. Specifying the lag structure. A lag space is constructed to represent the delayed effects of exposure across the study period. The lag dimension is independent of the time axis and may reflect different time scales depending on the study design. For example, Hb concentrations measured in the first and second trimesters may have distinct effects on the risk of LBW. Modeling the lag space allows us to assess how the timing of Hb measurement influences the outcome.

Step 3. Defining the cross-basis function. A cross-basis is specified by combining two basic functions, which model the shapes of exposure-outcome relationship and non-linear effects across lags (i.e. lag-outcome relationship). Based on the specified cross-basis, we can create an individual-specific Hb profile accounting for both the Hb trajectory and its lag effects. For example, the cross-basis can model how a 5 g/L increase in Hb is associated with LBW risk in early vs. late pregnancy, thereby illustrating differences in susceptibility windows across gestation.

We relaxed the assumption of a linear exposure–outcome relationship and used distributed lag non-linear model (DLNM) ^28^ to estimate the time-varying association between Hb concentration and birth outcomes. Given that the maximum gestational age in our study was 39 weeks, exposure periods were restricted to 1–39 gestational weeks. Because the functions (in both exposure and lag dimensions) and the detailed specifications such as the number and placement of knots—were unknown a priori, we selected the model with the smallest Akaike Information Criterion (AIC) (Supplementary Table S1). Subsequently, we used the estimated Hb exposure profile as predictors in a logistic regression model to obtain effect estimates within specific gestational windows. The logistic regression was constructed using a constrained structure of coefficients, representing the effect of each time point while incorporating the assumed lag–outcome relationship defined by the DLNM framework.

#### Generalized additive mixed model (GAMM)

This approach reverses the roles of Hb and birth outcomes, in which Hb is modeled as a function of time, while the outcome serves as a binary predictor ^29^. The model is specified as:

$$X_{ij}=\beta_{0}+b_{0i}+f_{1}\left(t_{ij}\right)\left(1-Y_{i}\right)+{\;f}_{2}\left(t_{ij}\right)Y_{i}+\epsilon_{ij}$$

where $\beta_{0}$ is the fixed intercept and $b_{0i}$ is the subject-specific random intercept, and $f_{1}\left(t_{ij}\right)$ and $f_{2}\left(t_{ij}\right)$ are smooth functions of gestational age that are allowed to differ by outcome status. The error term $\epsilon_{ij}$ is assumed to be normally distributed with mean 0. GAMM accounts for the longitudinal trend in Hb in relation to the outcome, allowing the association to be modeled parametrically or non-parametrically. Using this reverse modeling strategy, GAMM estimates difference in Hb trajectories according to outcome status. In our analysis, we fit the model with unconstrainted smooth functions, while limiting the degrees of freedom to prevent overfitting and to ensure trajectory reflected the commonly observed U-shape pattern of Hb across pregnancy ^30^. Predicted Hb values were then calculated across gestational age for mothers with and without PTB, LBW, or SGA, respectively.

#### Group-based trajectory modelling (GBTM)

GBTM can be used to identify subgroups of women with similar Hb trajectories during pregnancy ^31^ and to evaluate the association between these trajectory patterns and birth outcomes^14^. GBTM is a parametric finite mixture modeling approach that estimates trajectories by fitting polynomial functions of gestational age to Hb concentrations ^31^.

We first compared linear and quadratic mixed-effects models for Hb over gestational age and selected the better one based on model fit criteria. Next, latent class mixed models were fitted assuming different numbers of trajectory groups (e.g., one group representing a single trajectory for the entire population, two groups representing two distinct Hb trajectories, etc.). The optimal number of groups was determined by model fit criteria, with the additional requirement that each group contain at least 5% of participants ^32^.

After identifying the trajectory groups, each woman was assigned to the most likely group based on posterior probabilities, yielding a categorical variable that captures distinct patterns of Hb change across pregnancy. This trajectory group variable was then used as a predictor in logistic regression models to examine associations with adverse birth outcomes. Alternatively, to account for uncertainty in group membership assignment, the posterior probabilities of group assignment can also be incorporated as continuous predictors in logistic regression analyses.

## Results

### Participant characteristics and birth outcomes

Table 1 summarizes the characteristics of the women included in this study stratified by the number of Hb measurements. The majority (90%) of women had 2 Hb measurements (one at enrollment in the first or second trimester, and the other one at the end of gestation), and the rest 10% had 3 Hb measurements. For participants with three visits, gestational age had median values of 10 weeks (IQR: 9–11), 24 weeks (21–26), and 32 weeks (30–33) at visits 1, 2, and 3, respectively. Most women with 3 Hb measurements came from Gambia and Vietnam. The total number of previous births (parity) and the proportion of each adverse birth outcome appeared to be different between women with 2 and those with 3 measurements. Overall, Hb decreased from early to mid-pregnancy and increased slightly in the third trimester (Figure S2). Among the 6,452 pregnant women, 407 (7%) had PTB, 1175 (19%) had LBW, and 1909 (31%) had SGA babies. A total of 52 women experienced PTB and delivered SGA and LBW babies (Figure S3).

### Logistic regression using residuals Hb

In this method, we aimed to find the effect of trimester-specific changes in Hb on birth outcomes. Based on our modeling, the residuals represent deviations from the mean change in Hb during the second and third trimesters. Odds ratios (ORs) derived from the logistic regression using standardized residuals reflect the effect of a one–standard deviation increase in the residual, allowing for the comparison of relative differences. As shown in Figure S4, the 95% CI include the null value across all birth outcomes, which suggests that the residuals were not significantly associated with the odds of having PTB, LBW, or SGA.

### Two-stage mixed effects model

Overall, mean Hb in the second and third trimesters were estimated to be 8.7 (95% CI [7.6, 9.85]) and 6.25 (95% CI [5.1, 7.45]) g/L lower, respectively, compared to the first trimester (Table 2, Stage 1). We then examined whether individual deviations from the average population level were associated with the adverse birth outcomes. However, neither the subject-specific intercept nor the trimester-specific slopes were associated with any adverse birth outcome (Table 2, Stage 2), indicating no evidence that individual-level variations in hemoglobin trajectories were related to LBW, PTB, or SGA.

### Distributed lag non-linear model

**Figure 1.**
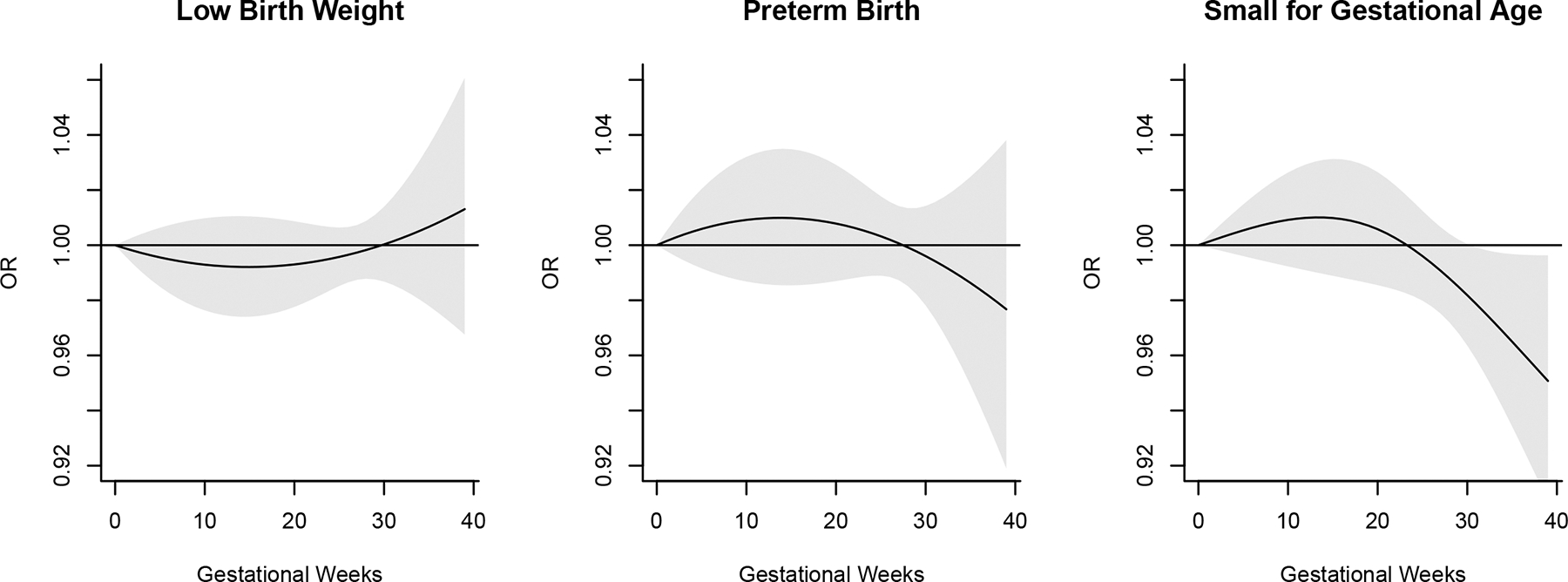
Estimated odds ratio (OR) of adverse birth outcomes associated with 5 g/L increase in Hb concentrations across gestation using distributed lag non-linear model assuming week-specific effects. The gray area indicates 95% confidence band (N = 676).

The primary objective of this method was to capture the time-varying association between Hb and birth outcomes and to estimate the timing of effects. By plotting the ORs across gestational ages, we were able to identify specific time windows during which the association changed. Figure 1 shows the estimated ORs of having LBW, PTB, and SGA associated with a 5 g/L increase in Hb from the reference value of 110 g/L across gestational age (in weeks). An increase in Hb concentration during the later stage of the third trimester (after week 30) appeared to be associated with lower odds of SGA. This suggests a gestational window–dependent effect of Hb.

### Generalized additive mixed model

**Figure 2.**
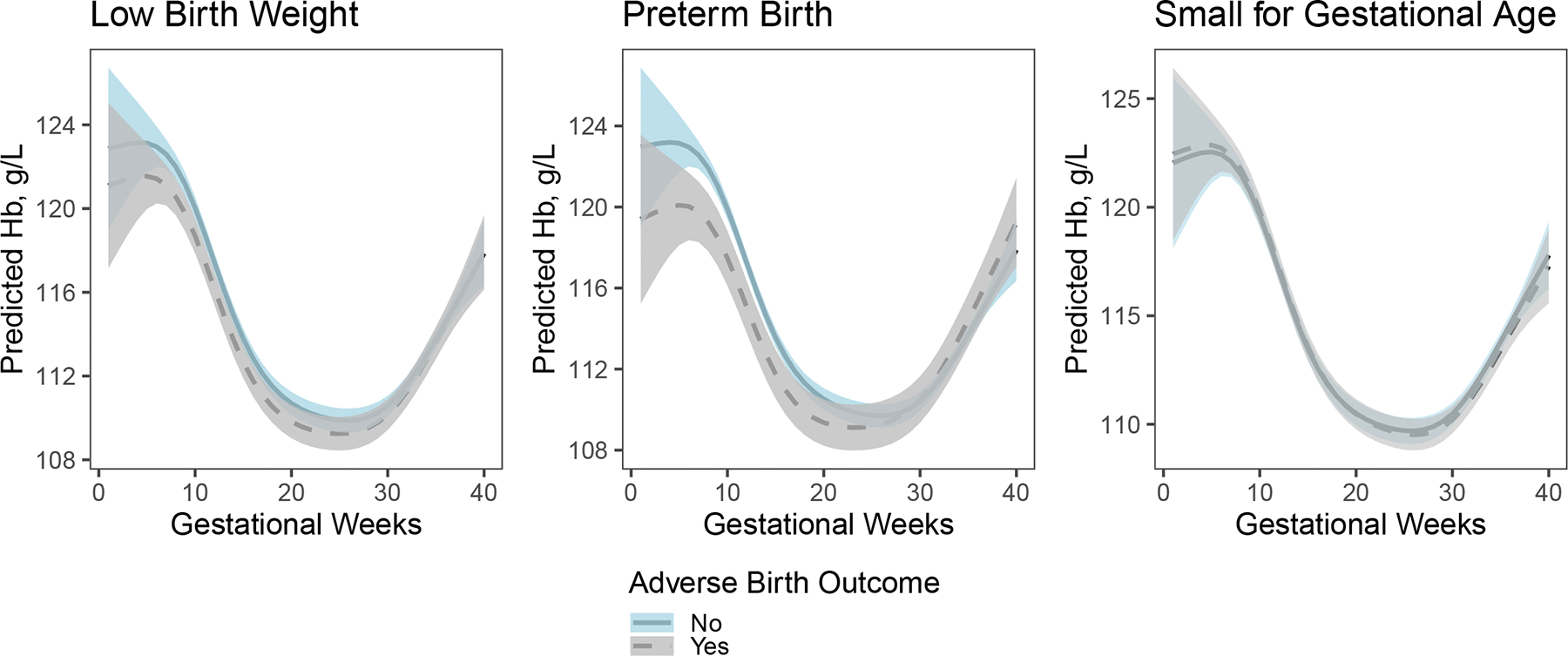
Predicted Hb and the corresponding 95% confidence bands across gestation using generalized additive mixed models (GAMM) in pregnant women with and without adverse birth outcomes (N=6,452).

The primary goal of the method was to model and compare the continuous trajectories of hemoglobin (Hb) across gestation between women with and without adverse birth outcomes, while accounting for nonlinear trends and within-subject correlation. Figure 2 shows smoothed Hb trajectories across gestation by adverse birth outcome status. The estimated degree of freedom (EDF) for the differences between outcome groups were respectively 2, 2.12, and 2 for LBW, PTB and SGA, indicating nonlinear differences in Hb trajectories.

For LBW and SGA, the 95% confidence bands of Hb concentrations for women with and without adverse birth outcomes overlapped throughout most of gestation, suggesting no statistically significant differences in predicted Hb concentrations. In contrast, women who delivered preterm infants consistently had lower estimated Hb before 20 weeks of gestation, compared to those without PTB.

### Group-based trajectory modeling (GBTM)

**Figure 3.**
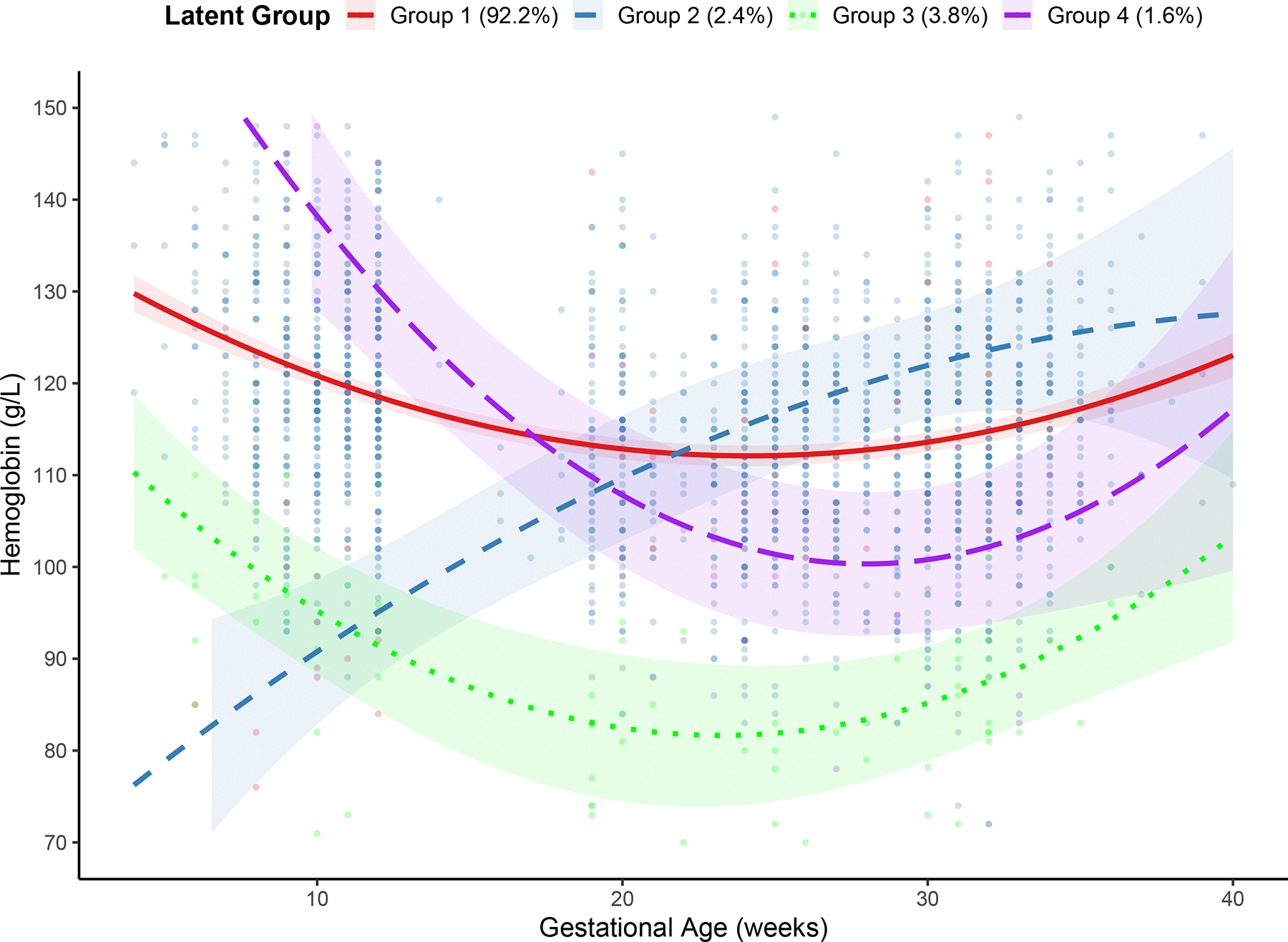
Observed and model-predicted hemoglobin Hb trajectories during pregnancy identified by group-based trajectory modeling. Lines represent model-predicted mean trajectories, and shaded areas indicate 95% confidence intervals (N = 676).

The primary goal of this method was to identify and characterize distinct latent trajectory groups of hemoglobin (Hb) across gestation. To examine Hb trajectories, we fitted both linear and quadratic specifications of gestational age and selected the quadratic model according to model fit criteria. Trajectory models with 1 to 7 groups were compared, and 4-group model was selected (Table S2). The observed and predicted Hb trajectories are shown in Figure 3.

Group 1 comprised the majority of participants (N = 623, 92.2%). This group exhibited a mild decline in Hb during mid-gestation followed by stabilization in late pregnancy. The remaining groups represented small and distinct subpopulations with their unique Hb trajectories. No significant associations were observed between trajectory group membership and the likelihood of adverse birth outcomes.

## Discussion

By examining the association between maternal Hb during pregnancy and birth outcomes using the BRINDA pregnancy data, our study illustrates statistical methods that can be used to model repeated measurements of a predictor in relation to a binary outcome in nutrition research. This work highlights how different analytical approaches can offer complementary perspectives on the same research question, thereby enriching interpretation and guiding more targeted recommendations and interventions to improve maternal and fetal health. No single method can be considered definitive, especially without adjusting for additional covariates. Researchers should be aware that conclusions about hemoglobin and birth outcomes may be highly sensitive to modeling decisions and select statistical strategies best suited to their study aims and data structure.

Many pregnancy and birth outcome studies in nutrition often conduct analysis stratified by time (e.g., trimester). However, these analyses often fail to leverage the longitudinal data from repeated measurements throughout pregnancy, potentially overlooking critical periods during which interventions may be most effective. While various statistical methods these study designs have been reviewed (Chen et al., 2015), we extend this work by incorporating DLNMs, which estimation of time-specific effects and identification of biomarker-lag-response relationships. To facilitate implementation, we also provide a sample dataset and R code for applying these models in other research contexts.

In the following discussion, we outline the strengths and limitations of each statistical method for modeling repeated Hb measurements in relation to birth outcomes (Table 3). Using residual Hb in a logistic regression framework incorporates all repeated measurements in one model, effectively addressing multicollinearity. The core inference of this method is the conditional effect. It tests if the fluctuation in Hb concentrations predict birth outcomes. This approach yields relatively robust estimates for each Hb measurement while controlling for Hb measured at other time points. However, it primarily captures the effects of individual measurements and does not account for temporal changes in Hb, which may have a greater influence on birth outcomes than absolute concentrations at specific time points.

The core inference of two-stage mixed-effects model is the association between individual-level trajectory and risk of birth outcomes. This method focuses on using individual’s deviation in intercept and slope from population average to predict birth outcomes. This approach can accommodate non-linear trends in Hb by incorporating smooth function of Hb over time in Stage 1 or higher-order terms as predictors in Stage 2. A notable limitation, however, is that the estimates derived from Stage 1 are treated as observed values in Stage 2, which may lead to biased estimates if their uncertainty is ignored.

DLNM is a powerful method to estimate Hb trajectory throughout pregnancy and its non-linear relationship with the probability of adverse birth outcomes. The most unique inference of the DLNM is its focus on pinpointing critical windows for potential interventions, enhancing our understanding of how the effects of Hb may vary across pregnancy. Unlike other methods that focus on aggregate effects, this approach identifies that Hb’s effect may be sensitive to a specific time window rather than across the entire trimester. However, the results derived from DLNM are contingent upon strong assumptions regarding the non-linear relationship between Hb and outcomes, as well as the distribution of lag effects. Violations of these assumptions can introduce bias into the findings. To evaluate these assumptions effectively, a diverse range of DLNMs is essential to evaluate different modeling choices. Regression diagnostics, such as residual and partial autocorrelation plots, provide investigators with critical insights (Gasparrini et al., 2010). Additionally, applying DLNM requires a large and complete dataset for accurate estimation, especially when the model incorporates numerous predictors or lag terms. Lastly, while DLNM focuses on elucidating the impact of changes in Hb on outcomes, it does not provide insights into the implications of actual Hb concentrations.

GAMM specifies a smooth function of time and evaluates the effects based on individual trajectories by reversing the roles of the dependent and independent variables. The primary goal of the GAMM is the visualized comparison of the actual Hb trajectories over time. While GAMM relaxes the linearity assumption and is relatively straightforward to implement, it does not yield direct effect estimates. Consequently, GAMM is primarily a valuable tool for visualization, and additional analyses are recommended to quantify associations.

The essential inference of GBTM is to identify distinct longitudinal patterns of Hb, allowing subgroups with different trajectories to be captured across pregnancy. The latent trajectories can be modeled with linear, quadratic, cubic, or higher-order polynomial functions. The finite mixture modeling approach can accommodate participants with differing numbers of measurements or irregular measurement timing. Each individual has a posterior probability of belonging to each trajectory group, which can be used to account for uncertainty in downstream analyses. Regardless, the number of groups and the polynomial order for each trajectory must be pre-specified or chosen via model fit criteria. Different choices can lead to different groupings, making results somewhat subjective. Also, small sample sizes or few repeated measures can lead to unstable group assignments or unreliable trajectory estimates. A reasonably large sample size with multiple measurements per subject is recommended, as this improves the accuracy of trajectory estimation, especially for nonlinear trends.

In summary, researchers are encouraged to explore multiple approaches and choose methods that best align with their research objectives, data structure, and characteristics of Hb measurements. However, a key limitation of this study is that the findings are based on the available data. Since potential confounders and effect modifiers were not accounted for, the results may not be fully generalizable. By demonstrating the application of these methods to model Hb in relation to birth outcomes, this study provides valuable insights into the strengths and limitations of different statistical approaches for analyzing time-varying biomarkers or other longitudinal predictors in pregnancy research. Ultimately, this study illustrated that the inference of adverse birth outcome risks is not a unique finding but is closely related with the assumptions and temporal scales of chosen statistical approach.

## Supplementary Material

Supplementary document

## Figures and Tables

**Table 1.** Summary of characteristics of pregnant women stratified by number of hemoglobin (Hb) measurements

	Women with 2 Hb measurements	Women with 3 Hb measurements	Overall
	(N=5,776)	(N=676)	(N=6,452)

Age (yrs)	25.1 (5.80)	26.5 (4.92)	25.3 (5.71)
Pre-pregnancy body mass index (kg/m^2^)	24.5 (4.33)	23.0 (4.71)	24.5 (4.35)
Parity	1.96 (1.57)	3.14 (2.24)	2.02 (1.63)
***Birth outcome***			
Low birth weight	1140 (20.1%)	35 (5.3%)	1175 (18.6%)
Preterm birth	387 (6.8%)	20 (3.1%)	407 (6.4%)
Small for gestational age	1805 (32.4%)	104 (17.0%)	1909 (30.9%)

Mean (standard deviation) or N (%) are presented. Parity includes live births and stillbirths.

**Table 2.** Results of linear mixed model (Stage 1) to investigate the association between trimester and hemoglobin and logistic regression (Stage 2) to examine the associations between subject-specific random intercepts and random slopes and birth outcomes (N=676)

*Stage 1: Associations between trimester and hemoglobin*						
Fixed Effect			Estimate		95% CI	

**Intercept (First Trimester)**			119.7		(118.6, 120.8)	
**Second vs. First Trimester**			−8.7		(−9.85, −7.6)	
**Third vs. First Trimester**			−6.25		(−7.45, −5.1)	

*Stage 2: Associations between subject-specific random intercept and random slope derived from Stage 1 and birth outcome*						
	Low birth weight		Preterm birth		Small for gestational age	

Random Effect	OR	95% CI	OR	95% CI	OR	95% CI

Intercept	0.926	(0.759, 1.131)	1.002	(0.994, 1.009)	0.932	(0.824, 1.054)
2^nd^ Trimester slope	0.672	(0.157, 2.874)	0.997	(0.942, 1.055)	1.038	(0.423, 2.547)
3^rd^ Trimester slope	1.383	(0.431, 4.439)	0.997	(0.953, 1.043)	0.940	(0.459, 1.926)

In stage 2, estimated odds ratios (OR) and 95% confidence interval (CI) corresponding to 5 g/L increase in Hb are shown.

**Table 3.** Advantages and limitations of statistical methods for modeling repeatedly measured biomarkers in relation to a binary outcome

Method	Primary goal	Data requirement	Advantages	Disadvantages
Residual Hb in logistic regression	• Isolates deviations from the expected Hb trajectory and tests their association with outcomes.	• At least two measurements per subject to compute deviations.	• Incorporates all repeated measurements in one model • Addresses multicollinearity • Relatively straightforward to implement	• Requires complete dataset • Focuses on individual measurements rather than trajectories, which may be more relevant for outcomes
Two stage mixed effects model	• Stage 1 models individual Hb trajectories, Stage 2 uses trajectory parameters (intercept/slopes) to predict outcomes.	• Requires multiple repeated measurements per subject to estimate slopes/intercepts reliably.	• Flexible modeling of biomarker pattern over time in Stage 1 • Accounts for individual-specific Hb trajectories	• Uncertainty from Stage 1 is not incorporated in Stage 2, which may lead to biased results
Distributed lag non-linear model	• Identifies the window of vulnerability where Hb impacts risks.	• Needs repeated measures at sufficient temporal resolution to model lag effects. The number and spacing of measurements affects power to detect critical windows.	• Captures nonlinear exposure–response relationships • Identifies critical windows (lag effects) • Time-specific effect estimation	• Requires more complex modeling and interpretation • May need substantial sample size and repeated measures
Generalized additive mixed model	• Estimates nonlinear trajectories between groups and accounts for repeated measures	• Requires repeated measurements; can accommodate unbalanced or irregularly timed data.	• Allows flexible, smooth modeling of time • Handles repeated measures data by incorporating random effects • Works with subjects who have unequal numbers of repeated measures or irregular timing of observations	• Not temporally logical • Does not provide direct effect estimates • Primarily a visualization tool; further analysis needed
**Group-based trajectory model**	• Identifies latent subpopulations with distinct longitudinal patterns.	• Requires repeated measures per subject, and enough observations per trajectory group for stable estimates.	• Identifies latent trajectory subgroups • Handles unbalanced or sparse repeated measures	• Results sensitive to model specification (number of groups, polynomial order) Cluster selection can be subjective • May yield very unbalanced groups • Requires reasonably large sample size for stable estimates

## Data Availability

Data described in the manuscript, code book, and analytic code will be made available upon request pending approval from the BRINDA steering committee and country.
