# Supplementary material for: Modeling the Association Between Repeated Measures of Hemoglobin During Pregnancy and Adverse Birth Outcomes
Source: J Nutr. Author manuscript; Available in PMC 2026 May 20. (PMC13185003; doi:10.1016/j.tjnut.2026.101511)
Supplement: Supplementary document [file NIHMS2167439-supplement-Supplementary_document.docx]

Modeling the Association Between Repeated Measures of Hemoglobin During Pregnancy and Adverse Birth Outcomes

Jiaxi Geng, Ziwei Zhang

***Supplementary Materials***

**Table S1**. Summary of the model selection process based on different assumption on the Hb function f(x) and lag function w(l) using distributed lag nonlinear model (DLNM)

| DLNMs |  |  |  |  | AIC |  |
| --- | --- | --- | --- | --- | --- | --- |
|  | f(x) | w(l) | df | LBW | PTB | SGA |
| Model 1 | Linear | Quadratic B-spline w intercept | 3 | 280.49 | 184.97 | 280.49 |
| *Model 2 | Linear | Quadratic B-spline w/o intercept | 2 | 278.69 | 184.21 | 278.48 |
| Model 3 | Linear | Quadratic B-spline w/o intercept (knots at 20) | 3 | 280.49 | 184.97 | 280.49 |
| Model 4 | Linear | Cubic B-spline w/o intercept | 3 | 280.49 | 184.97 | 280.49 |
| Model 5 | Linear | Natural spline w intercept (knots at 20) | 3 | 280.49 | 184.97 | 280.49 |
| Model 6 | Linear | Natural spline w/o intercept (knots at 20) | 2 | 278.50 | 184.34 | 278.50 |
| Model 7 | Linear | Natural spline w/o intercept (knots at 13, 29) | 3 | 280.49 | 184.97 | 280.49 |
| Model 8 | Quadratic B-spline (knots at 110) | Natural spline w intercept (knots at 20) | 12 | 283.99 | 199.37 | 283.99 |
| Model 9 | Quadratic B-spline (knots at 110) | Natural spline w/o intercept (knots at 20) | 8 | 279.03 | 195.20 | 279.03 |
| Model 10 | Cubic B-spline (knots at 110) | Natural spline w intercept (knots at 20) | 12 | 283.99 | 199.37 | 283.99 |
| Model 11 | Cubic B-spline (knots at 110) | Natural spline w/o intercept (knots at 20) | 8 | 279.03 | 195.20 | 279.03 |

Abbreviations: df, degree of freedom; AIC, Akaike Information Criterion; LBW, low birth weight; PTB, preterm birth; SGA, small for gestational age; B-spline, basis spline

* Selected model

**Table S2.** Comparison of group-based trajectory models and summary of latent class results. Models with 1–7 trajectory groups were evaluated using model fit criteria (e.g., Akaike and Bayesian Information Criterion)

| Model /  # of groups | AIC | BIC | % in class 1 | % in class 2 | % in class 3 | % in class 4 | % in class 5 | % in class 6 | % in class 7 |
| --- | --- | --- | --- | --- | --- | --- | --- | --- | --- |
| Linear | 16148.86 | 16171.32 | - | - | - | - | - | - | - |
| Quadratic | 16026.79 | 16054.86 | - | - | - | - | - | - | - |
| 1 | 16011.97 | 16034.55 | 100.00 | - | - | - | - | - | - |
| 2 | 15965.98 | 16006.63 | 2.07 | 97.93 | - | - | - | - | - |
| 3 | 15956.00 | 16014.71 | 2.37 | 96.01 | 1.63 | - | - | - | - |
| 4 | 15916.75 | 15993.52 | 2.37 | 92.16 | 3.85 | 1.63 | - | - | - |
| 5 | 15938.53 | 16033.37 | 2.96 | 91.12 | 0.30 | 2.81 | 2.81 | - | - |
| 6 | 15892.43 | 16005.34 | 3.25 | 4.44 | 0.30 | 83.43 | 3.70 | 4.88 | - |
| 7 | 15891.83 | 16022.80 | 6.36 | 3.40 | 3.85 | 0.30 | 78.55 | 1.48 | 6.07 |


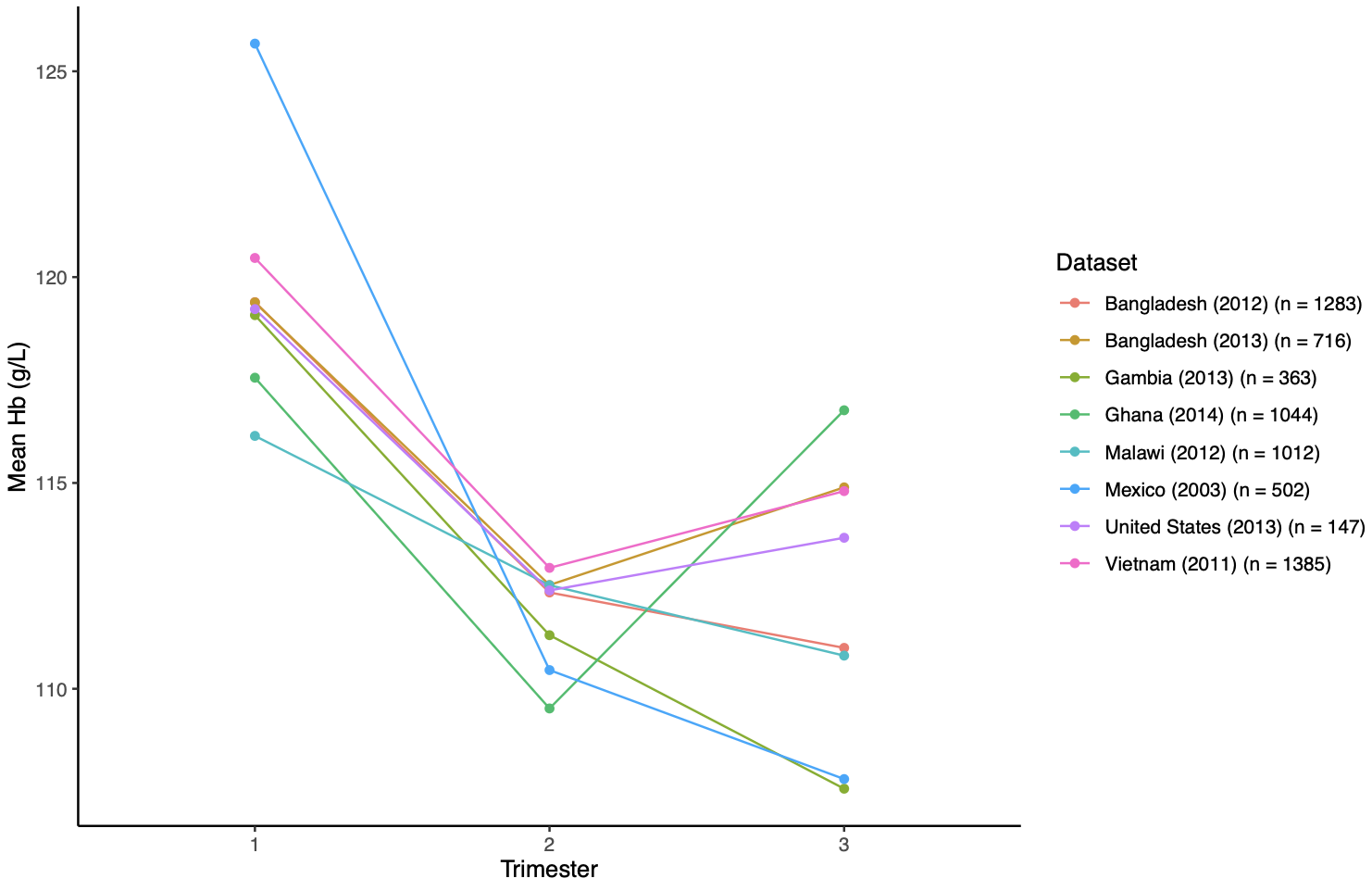


**Figure S1.** M*ean Hemoglobin (Hb)* concentration*s by trimester across included datasets*


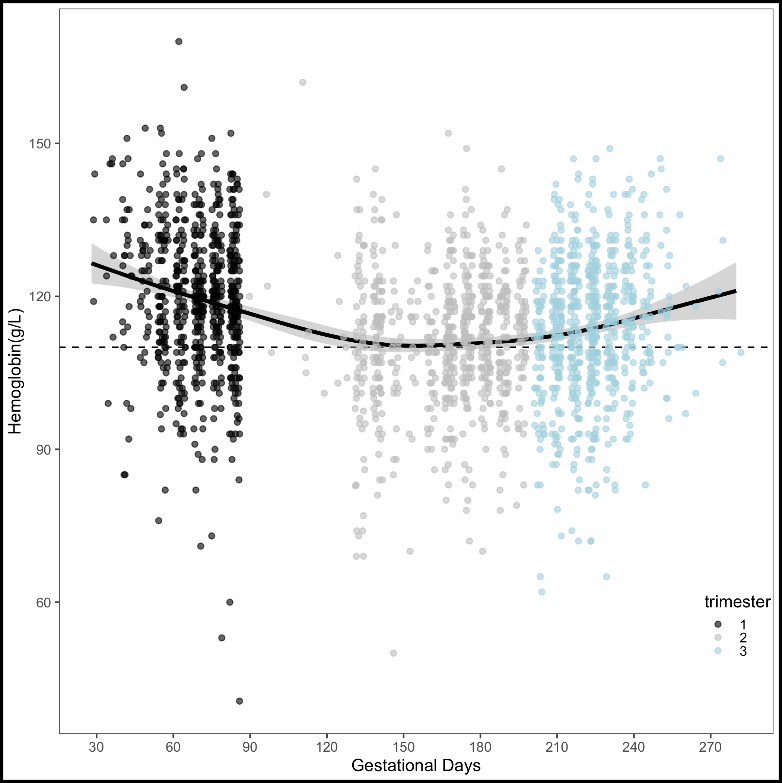


**Figure S2.** Scatterplot of Hb across gestational days with a smooth spline and 95% confidence band.


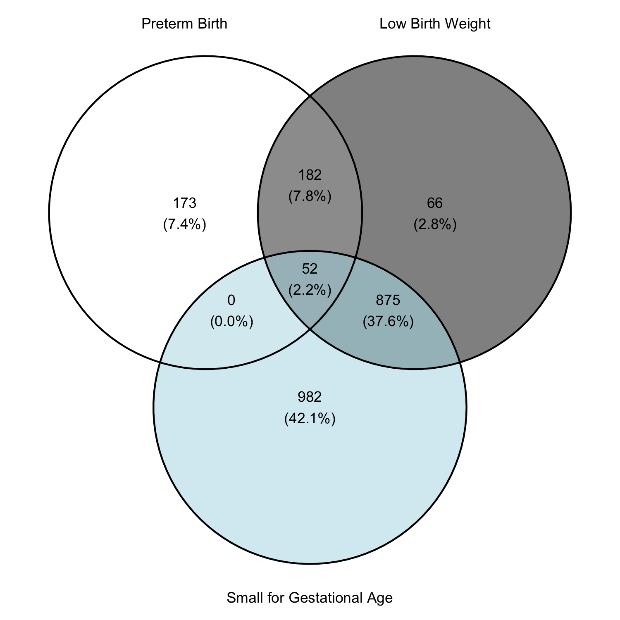


**Figure S3.** Number of women with adverse birth outcomes (total N=6,452).


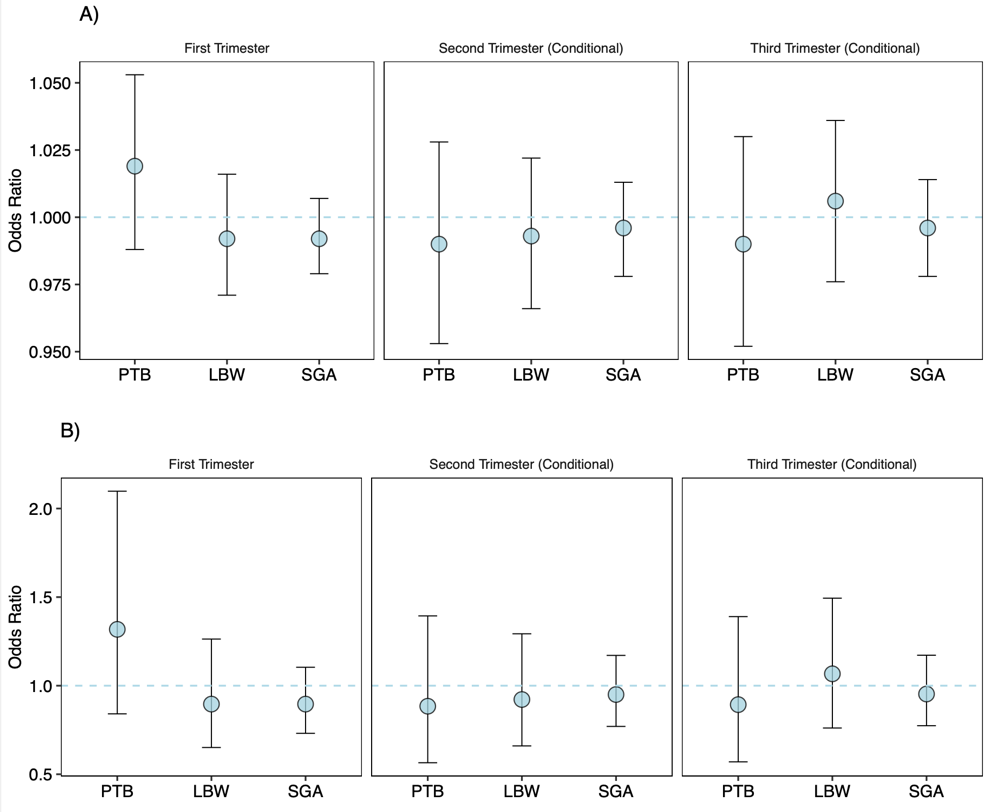


**Figure S4.** Odds ratios and the corresponding 95% confidence intervals of having preterm birth (PTB), low birth weight (LBW), and small-for-gestational age (SGA) outcomes. (A) Models using trimester-specific hemoglobin (Hb) values adjusted for earlier measurements. (B) Models using standardized trimester-specific Hb values adjusted for earlier measurements. "Conditional" refers to Hb values adjusted for earlier measurements. The residuals represent how much a woman's Hb deviated from the average change during the second and third trimesters.
